# Aqueous Humor Cytokines in Idiopathic Epiretinal Membrane: Correlation with Disease Severity

**DOI:** 10.3390/diagnostics14161797

**Published:** 2024-08-16

**Authors:** Tommaso Torresin, Angelo Greggio, Rino Frisina, Lorenzo Motta, Irene Gius, Giulia Midena, Edoardo Midena

**Affiliations:** 1 Ophthalmology Unit, Department of Neuroscience, University of Padova, 35128 Padova, Italy; tommaso.torresin@gmail.com (T.T.); angelo.greggio@gmail.com (A.G.); lorenzo.motta@unipd.it (L.M.); 2 Ophthalmology Unit, Surgery Department, Piacenza Hospital, 29121 Piacenza, Italy; frisinarino@gmail.com; 3 Department of Ophthalmology, SS Giovanni and Paolo Hospital, 30122 Venice, Italy; irene.gius.studente@gmail.com; 4 IRCCS—Fondazione Bietti, 00198 Rome, Italy; giulia.midena@fondazionebietti.it

**Keywords:** cytokines, proteomics, epiretinal membrane, aqueous humor

## Abstract

Background: To analyze the concentration of aqueous humor (AH) cytokines in eyes with idiopathic epiretinal membrane (iERM) and to investigate their potential correlation with disease severity. Methods: Retrospective cross-sectional case-control institutional study. A total of 16 eyes of 16 iERM patients and 14 eyes of 14 age-matched healthy controls were enrolled. AH samples were analyzed for various biomarkers using a glass-chip protein array. Cytokines associated with inflammation, fibrosis, angiogenesis, and glial signal transduction were quantified. Results: Significant differences in cytokine concentration were observed between the iERM group and controls, with 19 cytokines elevated in the iERM group (among them IL-6, IL-8, PDGF-AB, PDGF-BB, TGFB-1, TGFB-2, TGFB-3, VEGF A, VEGF C, VEGF D, *p* < 0,05, 95% confidence interval). Correlation analysis revealed associations between cytokine levels and iERM severity. Notably, stages 2, 3, and 4 of iERM demonstrated increased levels of various biomarkers. Conclusions: This study provides insights into the complex molecular interactions underlying iERM pathogenesis, describing a correlation between neuroinflammation and iERM severity.

## 1. Introduction

Epiretinal membrane (ERM) is a mainly retinal disorder characterized by fibrocellular membrane formation onto the retina’s surface over the inner limiting membrane (ILM), with a prevalence of 2.2% to 28.9% [1,2,3]. ERM is a commonly idiopathic condition (iERM) but can also result from trauma, chronic inflammatory diseases, intraocular surgery, or retinal detachment [4]. ERM often leads to reduced visual acuity and metamorphopsia [5]. High-resolution spectral domain coherence tomography (SD-OCT) plays a crucial role in the diagnosis and severity assessment of this disease [1,2,3,4,5,6,7], allowing for the visualization of retinal morphological alterations induced by iERM. Theories regarding the development of idiopathic ERM suggest that cellular proliferation, inflammation, and vitreous traction play significant roles. The proliferation of retinal glial cells and myofibroblasts can contribute to membrane formation, while inflammatory cytokines and chemokines may exacerbate this process. Additionally, tractional forces from the vitreous can cause further disruption and stimulation of membrane formation on the retinal surface [6,8,9,10,11,12].

However, in the last decades, the quantification and characterization of the exact intraocular molecular changes induced by retinal diseases has been gaining more and more importance to better understand their underlying pathophysiology. This may be obtained by sampling and analyzing ocular fluids, in particular aqueous (AH) or vitreous humor (VH). Sampling AH instead of VH is safer, repeatable, and less invasive, as previously reported [13,14], and the precise correlation of cytokine concentration profiles between AH and VH has been demonstrated [15,16]. Previous studies explored the inflammatory mediators’ link to iERM, but research into the presence of a potential correlation between intraocular cytokines and iERM severity is lacking [17,18,19,20].

In this study, we aimed to analyze the concentration of cytokines in the AH of eyes affected by iERM, comparing them to healthy eyes. Additionally, we investigated the relationship between these biomarkers and the severity of iERM using SD-OCT parameters in order to explore the role of neuroinflammation in the pathogenesis and progression of iERM.

## 2. Materials and Methods

This was a cross-sectional case-control study with prospective enrollment performed at the Ophthalmology Department of the University of Padova and AH examination at the laboratory of the Bietti Foundation. Patients affected by iERM were consecutively enrolled between June and December 2020. Informed consent was obtained from all patients, and the study adhered to the principles outlined in the Declaration of Helsinki. The Ethics Committee of the Azienda Ospedale Università di Padova approved the study (Protocol No. 3194/AO/14). Participants included in the study were male and female subjects aged 18 years or older who had been diagnosed with iERM and had retinal images of adequate quality. Patients were excluded if they had any systemic or ocular comorbidities or were receiving any topical or systemic treatments that could potentially affect ocular cytokine levels (e.g., diabetes mellitus, rheumatologic diseases, proliferative vitreoretinopathy, uveitis, glaucoma, local or systemic immunomodulatory or antiproliferative therapies). A control group, composed of age- and sex-matched patients undergoing cataract surgery with no ocular or systemic pathologies, was prospectively recruited during the same period.

### 2.1. Imaging and Disease Staging

All patients underwent a full ophthalmologic evaluation, including spectral domain-OCT (SD-OCT) (Nidek RS-3000, Nidek, Gamagori, Japan). An OCT horizontal linear scan passing through the fovea was used for the evaluation of iERM features. The disease stage assignment was performed by two independent masked operators (TT, AG) based on the classification proposed by Govetto et al. [3] as follows: (a) stage 1: presence of foveal pit and well-defined retinal layers; (b) stage 2: absence of the foveal pit but well-defined retinal layers; (c) stage 3: as in stage 2 with presence of ectopic inner foveal layers; (d) stage 4: as in stage 3 but with disruption of retinal layers. In the case of disagreement, the case was discussed with a third operator (EM) until agreement was reached.

### 2.2. Sample Collection and Preparation for Analysis

Patients planned for vitrectomy underwent AH sampling immediately at the beginning of the surgical procedure, whereas subjects included in the control group underwent AH sampling at the time of cataract surgery. Each enrolled subject underwent the standardized protocol for preoperative preparation before intraocular surgery, which includes disinfection of the periocular skin with 10% povidone-iodine (ESO-JOD, ECOLAB, Agrate Brianza, Italy), irrigation of the conjunctival sac with 5% povidone-iodine (Oftasteril, Alfa Intes, Casoria, Napoli, Italy) and a copious washing with balanced saline solution. The AH (150–200 µL) was aspirated by paracentesis from the anterior chamber using a 30-gauge needle attached to an insulin syringe (1 mL). Following aspiration, the AH was collected by a second operator in a single microcentrifuge containing 10 µL of a protease inhibitor cocktail (Pierce Biotechnology, Rockford, IL, USA). The labeled microvials were rapidly stored at −80 °C. Total protein content was quantified with a digital spectrophotometer (NanoDrop; Thermo Fisher Scientific Inc, Waltham, MA, USA), and protein concentration was calculated using the linearized standard curve (bovine serum albumin) and A280 software (version 3.8.1). The AH samples were then processed by sonication (VibraCell; Sonics, Newton, CT, USA) and centrifugation to collect the clear supernatant (13,000 rpm/7 min). The subsequent specific biomarkers analyzed were chosen after revision of the existing literature to identify biomarkers that could be representative of each category, even considering practical and economic reasons [9,10,11,12,15,19,21,22,23,24,25,26,27,28,29,30,31,32].

### 2.3. Glass-Chip Protein Array Analysis

The biochemical biomarker analysis utilized a customized glass-chip protein array provided by RayBiotechTM (Norcross, GA, USA). Normalized and prediluted AH samples were loaded onto the array following the manufacturer’s protocol. The slides were incubated overnight at approximately 4 °C, then washed and treated with a biotinylated antibody mixture. This was followed by the addition of a cy3-streptavidin solution. All steps were performed on a special orbital motion shaker (Certomat II; Sartorius AG, Göttingen, Germany) using the hybridization and washing solutions supplied with the kit. Subsequent washing of the slides was performed with MilliQ water, after which they were centrifuged and scanned using a microarray scanner (Molecular Devices LLC, Sunnyvale, Silicon Valley, CA, USA). To ensure an appropriate Cy3/Cy5 ratio (specific signal to background signal), all slides were scanned according to previously validated parameters and procedures. Fluorescence signals were captured using the GenePix 4100 microarray scanner (Molecular Devices, LLC, Sunnyvale, Silicon Valley, CA, USA), operated with GENEPIX pro 3.0 software (Axon Instrument, Foster City, CA, USA). In the analysis of fluorescence values, the median value was used as the reference for each biomarker change, excluding the “tails” from the evaluation. An intra- and inter-assay coefficient of variability of less than 10% was maintained, and a signal increase of 1.5 times or a reduction to 0.65 times the baseline was considered adequate to ensure a signal distinguishable from the background. The fluorescence signals were then analyzed. Twenty-six biomarkers were quantified, including inflammatory interleukins (IL-6, 8, 12p40, 12p70); Osteopontine (OPN); Macrophage Inflammatory Protein (MIP)-3α; Glial Cell-Derived Neurotrophic Factor (GDNF); Monocyte Chemoattractant Protein (MCP)-1; Granulocyte-Macrophage Colony-Stimulating Factor (GM-CSF); Vascular Endothelial Growth Factor (VEGF) A, VEGF C, VEGF D; Tumor Growth Factor (TGF) β1, β2, β3; Growth Factor (PDGF)-AA, AB, BB; Macrophage-Derived Chemokine (MDC); CC Chemokine Ligand 17 (CCL17); chemokine (C-X-C motif) Ligand (CXCL1, 16); Fractalkine (CX3CL1); Macrophage Migration Inhibitory Factor (MIF); Erythropoietin (EPO); and Pigment Epithelium-Derived Factor (PEDF).

### 2.4. Immunoprecipitation and SDS-PAGE Analysis

For the direct immunoprecipitation (IP) technique, capture antibodies specific to the inwardly rectifying potassium channel (Kir) 4.1 were incubated with Pure Proteome Protein G Magnetic beads (15 μL; Millipore, Burlington, MA, USA). These antibodies were immobilized using a magnet to form the antibody–bead complex. The bead-bound antibodies were then added to the normalized protein samples (30 μg total protein). After a 2 h incubation, the captured proteins were washed and eluted in denaturing Loading Buffer. All steps were conducted under orbital shaking (Certomat II; Sartorius AG, Gottinga, Germany). Both the Loading Buffer and samples were preheated at 90 °C for 10 min before being loaded onto 4–12% precast SDS-PAGE gels (Bio-Rad Laboratories Inc., Hercules, CA, USA). Electrophoresis was carried out in a MiniProtean3 apparatus (Bio-Rad Laboratories Inc., Hercules, CA, USA) under reducing conditions at 120 V/frontline. Following electrophoresis, the bands were transferred to 0.22 μm membranes (Hybond; GE Healthcare, Buckinghamshire, UK) in a semidry Trans-Blotting apparatus (Bio-Rad Laboratories Inc., Hercules, CA, USA) at 12 V for 40 min. The membranes were stained with Sypro Ruby protein blot stain (Invitrogen, Waltham, MA, USA) to visualize and capture specific bands, following standard procedures. Immunoblotting with chemiluminescent detection was also performed to enhance visualization of the protein of interest. The optical density (OD) of the bands was measured using ImageJ software (ImageJ v1.43; NIH, https://imagej.net/, accessed on 1 October 2021). Data were saved as 8-bit TIFF files and exported for figure assembly using the Adobe Photoshop 2022 22.0.0 software release (Adobe Systems Inc., San Jose, CA, USA).

### 2.5. Enzyme-Linked Immunosorbent Assay

Glial fibrillary acidic protein (GFAP) levels were measured using a commercially available high-sensitivity enzyme-linked immunosorbent assay (ELISA) kit (NS830 from Millipore; Merck KGaA, Darmstadt, Germany), following the manufacturer’s instructions with minor modifications. In brief, prediluted samples and standard curves were further diluted in TBS buffer (20 mM Tris-Cl and 150 mM NaCl, pH 7.5) containing 3% bovine serum albumin, 5 mM EDTA, and 1X protease inhibitor cocktail. These were then loaded onto 96-well plates pre-coated with the capture antibody. Detection involved the use of biotin-conjugated antibodies and horseradish peroxidase-streptavidin, with the specific binding visualized using a ready-to-use tetramethylbenzidine (TMB) substrate (eBioscience, San Diego, CA, USA). The resulting colorimetric signal was measured using an ELISA reader at wavelengths between 490 and 560 nm (Sunrise; Tecan Group Ltd., Männedorf, Switzerland). The optical density (OD) values were normalized to total protein content determined using a NanoDrop spectrophotometer. GFAP concentrations were calculated using linearized standard curves provided with the ELISA kit. The assay’s standards ranged from 1.5 to 100 ng/mL, with a detection limit of 1.5 ng/mL.

### 2.6. Statistical Analysis

Data are presented as the mean ± standard deviation. The normality of the data distribution was assessed using the Shapiro–Wilk test. For each protein, comparisons of AH protein concentrations between eyes with iERM and control eyes were made using the *t*-test. Power analysis was conducted to determine the probability that the *t*-tests correctly reject the null hypothesis when it is false, using a significance level (alpha) of 0.05. The power of each test was calculated based on the observed means, standard deviations, and sample sizes of the iERM and Healthy Control (HC) groups. Linear regression analysis was conducted to determine whether the severity of iERM (stages 1–4) correlated with each biomarker level. Data analysis was performed using SAS statistical software (SAS 9.2; SAS Institute, Cary, NC, USA). A *p*-value of less than 0.05 was considered statistically significant. Pearson’s correlation coefficients greater than 0.80 were interpreted as indicating excellent correlation.

## 3. Results

A total of 16 eyes of 16 patients and 20 eyes of 20 healthy controls were enrolled. Overall, six healthy eyes were excluded from the control group due to the insufficient amount of AH sample. Therefore, 16 iERM patients (16 eyes) and 14 healthy controls (14 eyes) were considered for the analysis. The mean age was 72.2 ± 9.5 years in the iERM group and 75 ± 5.5 years in the controls, respectively (*p* = 0.36). There was no significant difference in gender distribution between the two groups (eight males (50%) and eight females (50%) in the iERM group, and six males (43%) and eight females (57%) in the control group, *p* = 0.69). According to the OCT-based classification described by Govetto et al., iERM was classified into four stages, and four eyes were comprised in each stage subgroup (iERM subgroups 1–4). Demographic and ophthalmological data of each subgroup are reported in Table 1.

**Figure 1:**
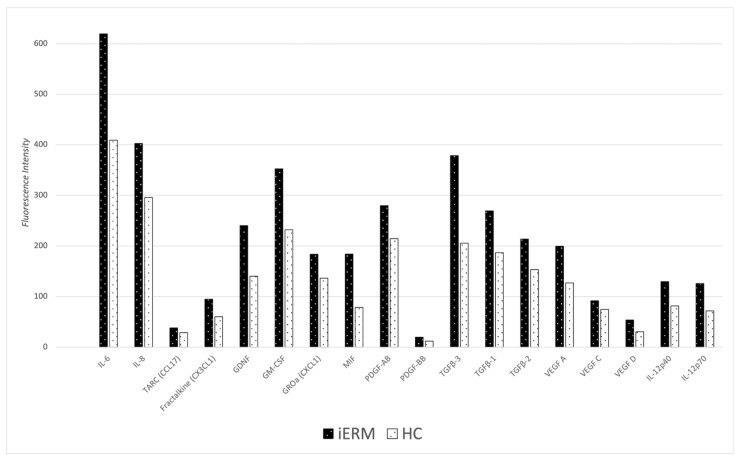
Comparison of significant cytokines concentration in iERM and Healthy Control (HC) groups. IL-6: Interleukin-6; IL-8: Interleukin-8; TARC (CCL17): Thymus and Activation-Regulated Chemokine; FRACTALKINE (CX3CL1): Fractalkine (C-X3-C Motif Chemokine Ligand 1); GDNF: Glial Cell Line-Derived Neurotrophic Factor; GM-CSF: Granulocyte-Macrophage Colony-Stimulating Factor; GROa (CXCL1): Growth-Regulated Alpha Protein (C-X-C Motif Chemokine Ligand 1); MIF: Macrophage Migration Inhibitory Factor; PDGF-AB: Platelet-Derived Growth Factor-AB; PDGF-BB: Platelet-Derived Growth Factor-BB; TGFβ-3: Transforming Growth Factor Beta-3; TGFβ-1: Transforming Growth Factor Beta-1; TGFβ-2: Transforming Growth Factor Beta-2; VEGF A: Vascular Endothelial Growth Factor A; VEGF C: Vascular Endothelial Growth Factor C; VEGF D: Vascular Endothelial Growth Factor D; IL-12p40: Interleukin-12 Subunit p40; IL-12p70: Interleukin-12 p70; MIF: Macrophage Migration Inhibitory Factor.

The concentration of cytokines in AH samples was initially compared between the iERM group and the control group, as shown in Table 2 and Figure 1.

**Figure 2:**
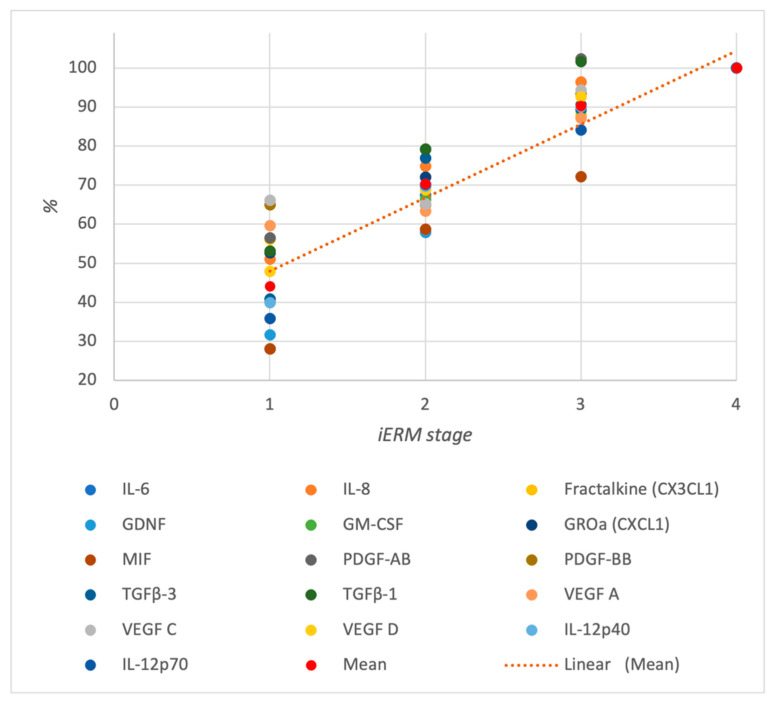
% normalized correlation between iERM stage and significant cytokine concentrations. Note the positive correlation between cytokine levels and the iERM stage, indicating that higher cytokine concentrations are associated with more advanced stages of iERM. IL-6: Interleukin-6; IL-8: Interleukin-8; GM-CSF: Granulocyte-Macrophage Colony-Stimulating Factor; IL-12p40: Interleukin-12 Subunit p40; IL-12p70: Interleukin-12 p70; MIF: Macrophage Migration Inhibitory Factor; TGFβ-1: Transforming Growth Factor Beta-1; TGFβ-2: Transforming Growth Factor Beta-2; TGFβ-3: Transforming Growth Factor Beta-3; VEGF A: Vascular Endothelial Growth Factor A; VEGF C: Vascular Endothelial Growth Factor C; VEGF D: Vascular Endothelial Growth Factor D; TARC (CCL17): Thymus and Activation-Regulated Chemokine (CCL17); FRACTALKINE (CX3CL1): Fractalkine (C-X3-C Motif Chemokine Ligand 1); GROa (CXCL1): Growth-Regulated Alpha Protein (C-X-C Motif Chemokine Ligand 1); PDGF-AB: Platelet-Derived Growth Factor-AB; PDGF-BB: Platelet-Derived Growth Factor-BB; GDNF: Glial Cell Line-Derived Neurotrophic Factor.

Subsequently, a further analysis was conducted specifically on the biomarkers that exhibited significant differences during the initial comparison. This subsequent analysis focused on comparing the individual iERM subgroups (1–4) with the control group. No significant differences in cytokines concentration were observed between the iERM 1 subgroup and the control group. However, the iERM 2 subgroup showed significantly higher levels of TARC (CCL17) (*p* = 0.001), Fractalkine (*p* = 0.003), MIF (*p* = 0.025), PDGF-BB (*p* < 0.001), TGF-β3 (*p* = 0.021), VEGF A (*p* = 0.019), VEGF D (*p* = 0.014), and IL-12p70 (*p* = 0.020) compared with the control group. The iERM 3 subgroup exhibited significantly higher levels of IL-6 (*p* < 0.001), IL-8 (*p* < 0.001), TARC (CCL17) (*p* = 0.014), Fractalkine (CX3CL1) (*p* = 0.022), GDNF (*p* = 0.007), GM-CSF (*p* < 0.001), GRO-a (CXCL1) (*p* = 0.003), MIF (*p* = 0.041), PDGF-AB (*p* < 0.001), PDGF-BB (*p* = 0.013), TGF-β1 (*p* < 0.001), TGF-β3 (*p* < 0.001), VEGF A (*p* = 0.029), VEGF C (*p* = 0.018), VEGF D (*p* = 0.003), IL-12p40 (*p* = 0.003), EPO R (*p* = 0.031), and IL-12p70 (*p* < 0.001) compared with the control group. Likewise, the iERM 4 group displayed significant differences in levels of IL-6 (*p* < 0.001), IL-8 (*p* < 0.001), TARC (CCL17) (*p* = 0.027), Fractalkine (CX3CL1) (*p* < 0.001), GDNF (*p* < 0.001), GM-CSF (*p* < 0.001), GRO-a (CXCL1) (*p* < 0.001), PDGF-AB (*p* < 0.001), PDGF-BB (*p* = 0.040), TGF-β1 (*p* < 0.001), TGF-β2 (*p* < 0.001), TGF-β3 (*p* < 0.001), VEGF A (*p* = 0.001), VEGF C (*p* < 0.001), VEGF D (*p* < 0.001), IL-12p40 (*p* < 0.001), EPO R (*p* < 0.001), and IL-12p70 (*p* < 0.001) compared with the control group. Furthermore, the correlations between cytokine levels in AH and iERM stage (1–4) were also examined, and the results are shown in Table 3, indicating several excellent positive correlations (Figure 2).

## 4. Discussion

iERM pathogenesis involves complex, not fully understood cellular and molecular processes. Recent research highlights the roles of inflammation and associated cytokines in iERM, but limited and conflicting proteomics data exist [17,18,19,20]. iERM is composed of various cell types, including Müller cells, astrocytes, hyalocytes, retinal pigment epithelium cells, fibroblasts, and myofibroblasts [9]. Notably, myofibroblasts, which mainly differentiate from resident fibroblasts, play a central role in collagen production and the subsequent contraction of iERM, leading to the characteristic retinal wrinkling typical of this disorder, while elevated levels of cytokines contribute to inflammation and fibrotic scar formation in iERM-affected eyes [21,25]. Elevated cytokine levels in iERM can arise from various sources and processes associated with the pathophysiology of this condition like inflammatory response and mechanical cellular stress and damage. Inflammatory cells, including macrophages and microglia, can release proinflammatory cytokines, such as IL-6, IL-8, TNF-α, and IL-1β. These cytokines contribute to the inflammatory cascade, leading to tissue damage and fibrosis characteristic of iERM [11,25]. However, the formation of epiretinal membranes causes cellular stress and damage to retinal cells, including Müller cells and retinal pigment epithelial cells. This cellular damage triggers the release of cytokines as part of the repair and remodeling processes. Cytokines such as TGF-β, PDGF, and VEGF are known to be involved in fibrosis, angiogenesis, and tissue remodeling, all of which are processes associated with iERM [33].

The results of our study confirmed significant differences in cytokine concentration between eyes with iERM and the control group. Among the 32 cytokines analyzed, 19 were found to be increased in the iERM group compared with controls, confirming the involvement of neuroinflammatory, angiogenic, and fibrotic pathways in the pathophysiology of iERM [11].

The classification of measured cytokines into proinflammatory, profibrotic, angiogenic, chemokines, and growth factors provides valuable insights into the specific pathways involved in iERM pathogenesis. By segregating cytokines based on their functional roles, this study offers a more detailed understanding of the complex molecular interactions contributing to disease development and progression.

Of particular interest is the observed correlation between levels of AH inflammatory mediators and the severity of iERM, as classified by Govetto et al.’s staging system, which classifies the condition into four distinct stages based on morphological changes, retinal distortion, inner foveal layer presence, and retinal layers disorganization [3].

Similar associations between disease severity and cytokine levels have been reported in other retinal disorders, such as diabetic retinopathy, where elevated levels of IL-6, IL-8, and VEGF were correlated with disease progression [34,35,36].

Although some studies analyzing intraocular cytokine concentration used eyes with iERM as control groups, their appropriateness remains insufficiently supported. A study conducted by Zandi et al. in 2016 revealed significantly elevated levels of cytokines in the VH of individuals with iERM compared with those with a macular hole, suggesting that a cautious approach is necessary when considering the suitability of eyes with iERM as a healthy control group [26].

Our results also confirm higher levels of cytokines in AH in iERM eyes compared with controls. However, it is important to note that in subgroup 1 of iERM, while cytokines were increased, they were not significantly different from the control group, perhaps due to a non-significant inflammatory response at this early stage or a localized effect still confined to the microenvironment of the diseased retina. This emphasizes the importance of separately considering stage 1 iERM eyes and suggests the possibility of using them as controls in certain contexts regarding proteomic studies on AH or VH. 

Inflammatory mediator involvement in iERM pathogenesis is supported by studies showing elevated proinflammatory cytokine levels, like IL-6, TNF-α, and IL-1β, in iERM eyes’ VH and AH [17,18,19,20]. These, along with growth factors like TGF-β and VEGF, promote proliferation, fibrosis, angiogenesis, and vascular permeability crucial for epiretinal membrane formation [8,11]. Chemokines such as MCP-1 and IL-8 also participate in attracting and activating immune cells to retinal tissue [11,37].

In our study, among the analyzed proinflammatory cytokines, the iERM group showed increased levels of IL-6 and IL-8, which correlated with disease severity. IL-8, predominantly secreted by fibroblasts and inflammatory cells, may enhance the fibrotic response and combine with TGF-β1 to regulate fibroblast differentiation, thus inducing fibrotic processes [38,39].

We also found significantly higher levels of GRO-α (CXCL1), MIF, PDGF-AB, and PDGF-BB in the iERM group compared with the control group. GRO-α (CXCL1) is a chemoattractant for neutrophils and a mediator of inflammation and angiogenesis [40], while PDGF-BB and PDGF-AB are both chemoattractants for fibroblasts and can induce them to secrete TGF-β, leading to fibrotic proliferation and ERM formation and development [41,42]. The presence of chemokines, such as TARC (CCL17) and Fractalkine (CX3CL1), in iERM eyes further supports the involvement of immune cell recruitment and neuroinflammation in the pathogenesis of iERM [43,44].

Of the analyzed profibrotic cytokines, our results demonstrate the involvement of TGF-β1, TGF-β2, and TGF-β3 in iERM formation. TGF-β3, however, correlates more strongly with the severity of iERM (Pearson’s coefficient = 0.85), whereas the correlation of TGF-β1 is borderline (Pearson’s coefficient = 0.79) and TGF-β2 (Pearson’s coefficient = 0.55) does not correlate significantly. TGF-β is known to play a critical role in fibrotic processes and extracellular matrix deposition [45]. This aligns with the pathological processes observed in the formation of iERM. According to a study by Minchiotti et al., it is reasonable to assume that TGF-β1 can target glial cells and stimulate them to transdifferentiate into myofibroblasts and activate their contractile actions [22]. A study by Iannetti et al. reported the role of TGF-β1 and TGF-β2 in the pathogenesis of iERM [27], and a previously reported study by Kohno et al. also demonstrated the importance of TGF-β2 in iERM contraction [12].

VEGF is one of the most extensively studied vitreoretinal growth factors among the proangiogenic molecules and has also been reported in iERM. Our results show that all VEGF isoforms (A, C, D), together with EPO R levels, were higher in eyes with iERM. In a study by Mandelcorn et al. [29], 85% of iERM stained positively for VEGF, and positive VEGF immunoreactivity of iERM was also found in a study by Chen et al. [30]. As previously reported, retinal glial cells produce VEGF, so these results are not surprising [9,46]. However, it is still challenging to understand why there are no blood vessels in iERM despite the presence of VEGF. Some authors have proposed two possibilities: either there are other cells in the iERM besides endothelial cells that are targeted by VEGF, or the presence of endothelial growth inhibitory factors, such as TGF-β, may prevent VEGF from exerting its angiogenic activity [47].

Furthermore, as glial cells are known to be one of the most important cellular components of iERM, it is important to understand the role of molecules involved in glial signal transduction. We observed elevated levels of molecules involved in glial signal transduction in the AH of eyes with iERM; however, Müller cells activation biomarkers (G-FAP, AQP4, and Kir 4.1) showed no significant difference in concentration between iERM eyes and the control group. 

Müller cells are known to play a central role in the pathophysiology of iERM [10]. Various cytokines and growth factors serve as autocrine and paracrine modulators, stimulating Müller cell proliferation, migration, collagen contraction, and transdifferentiation [48,49,50,51]. Notably, TGF-β has been identified as a key inducer of glial-to-mesenchymal transition in Müller cells. This transition involves the downregulation of Müller cell glial markers concomitant with the upregulation of profibrotic myofibroblast markers [31,32,52]. This supports our findings where we did not observe an increase in Müller cell markers in AH of iERM eyes.

GDNF and Fractalkine (CX3CL1) levels were instead elevated in the iERM group and correlated significantly with the severity of the disease. A study by Harada et al. [23] also showed that GDNF may be involved in the formation of iERM, while Fractalkine (CX3CL1) is known to be a key regulator of microglial function and a microglial chemoattractant which could cause microgliosis.

The findings of this study could have some important clinical implications. Identifying specific cytokine profiles associated with iERM severity could lead to the development of new diagnostic biomarkers for earlier detection and more accurate disease monitoring. Furthermore, correlating cytokine levels with SD-OCT parameters could help in creating personalized treatment plans, improving patient outcomes by tailoring treatments to individual disease characteristics.

However, it is important to acknowledge the study’s limitations. The relatively small sample size may limit the generalizability of the results. Additionally, the cross-sectional design of the study prevents the establishment of causal relationships between biomarkers and disease progression. To further validate and expand upon these findings, future research may involve larger prospective studies with well-defined control groups. Longitudinal studies could also be conducted to assess the dynamic changes in biomarker levels during iERM progression, providing deeper insights into the temporal relationship between biomarkers and disease severity. Another limitation of this study is the omission of vitreous samples in the determination of cytokine concentrations in patients who underwent vitrectomy. Analyzing vitreous samples could have provided valuable results by enabling a direct comparison of cytokine levels between the AH and VH. We decided to focus on the analysis of AH rather than vitreous samples to ensure the purity of the fluid collected. The insertion of trocars, necessary for VH sampling, involves mechanical manipulation of the ocular tissue, which could potentially alter cytokine levels. To avoid any influence from such procedures on the results, we chose to collect AH, a sample that more accurately reflects the physiological state without external interference. This decision is supported by the precise correlation of cytokine concentration profiles between aqueous humor and vitreous humor, as demonstrated in previous studies [15,16]. Despite this, we acknowledge that the inclusion of VH samples could have enhanced the robustness of our findings. Future studies should consider incorporating vitreous sampling to provide a more comprehensive analysis of cytokine levels in both ocular compartments.

## 5. Conclusions

In conclusion, our study provides valuable insights into the role of neuroinflammation in the pathogenesis and progression of iERM. To our knowledge, our study is the first to illustrate the AH cytokine concentration correlated with iERM disease severity. The identified correlations between biomarker levels and disease severity offer important insights into the underlying mechanisms driving iERM.

## Figures and Tables

**Table 1:** Demographic and ophthalmological data of subgroups.

	n	Age Mean ± SD (years)	Gender M/F	Mean Visual Acuity (LogMAR)
HC group	14	72.2 ± 9.5	7/7	0.37 ± 0.17
iERM subgroup 1	4	73.0 ± 6.2	2/2	0.20 ± 0.00
iERM subgroup 2	4	74 ± 1.2	3/1	0.40 ± 0.35
iERM subgroup 3	4	76.2 ± 1.0	1/3	0.43 ± 0.22
iERM subgroup 4	4	77 ± 3.8	2/2	0.50 ± 0.16

SD: standard deviation.

**Table 2:** Comparison of cytokines between iERM and Healthy Control group and their primary function.

Cytokine	Cytokine Primary Function	iERM Group Mean ± SD (16 Eyes)	HC Group Mean ± SD (14 Eyes)	*p*-Value	Statistical Power Value
IL-6 (FI)	PI	619.50 ± 280.17	408.93 ± 206.39	**0.026** ^†^	0.76
IL-8 (FI)	PI	402.72 ± 117.33	296.07 ± 100.39	**0.012** ^†^	0.79
MCP-1 (FI)	PI	572.69 ± 229.93	506.36 ± 252.15	0.461 ^†^	0.10
MDC (FI)	C	16.03 ± 3.64	15.00 ± 4.21	0.482 ^†^	0.10
TARC (CCL17) (FI)	C	38.09 ± 7.72	28.79 ± 8.75	**0.005** ^†^	0.85
MIP-3a (FI)	PI	61.28 ± 26.27	54.36 ± 19.09	0.412 ^†^	0.11
CXCL16 (FI)	C	440.19 ± 285.44	619.29 ± 457.81	0.220 ^†^	0.001
FRACTALKINE (CX3CL1) (FI)	C	94.56 ± 24.81	60.07 ± 23.13	**<0.001** ^†^	0.96
GDNF (FI)	GF	239.97 ± 108.01	140.43 ± 73.91	**0.006** ^†^	0.80
GM-CSF (FI)	PI	352.38 ± 128.33	231.79 ± 100.53	**0.008** ^†^	0.78
GROa (CXCL1) (FI)	C	183.56 ± 49.93	136.29 ± 38.92	**0.007** ^†^	0.79
MIF (FI)	PI	183.81 ± 91.86	78.42 ± 80.14	**0.031** ^†^	0.89
PDGF-AA (FI)	GF	81.47 ± 37.72	107.79 ± 78.40	0.267 ^†^	0.002
PDGF-AB (FI)	GF	279.72 ± 72.35	214.29 ± 63.06	**0.013** ^†^	0.81
PDGF-BB (FI)	GF	19.91 ± 5.98	12.07 ± 2.39	**<0.001** ^†^	0.99
OPN (FI)	PI	4983.16 ± 3762.79	5618.36 ± 1828.35	0.555 ^†^	0.007
TGFβ-3 (FI)	PF	378.69 ± 126.18	205.57 ± 105.72	**<0.001** ^†^	0.97
TGFβ-1 (FI)	PF	269.09 ± 76.93	186.71 ± 70.87	**0.005** ^†^	0.83
TGFβ-2 (FI)	PF	213.34 ± 53.04	153.07 ± 66.89	**0.012** ^†^	0.76
VEFG A (FI)	PA	199.56 ± 53.97	127.14 ± 48.84	**0.001** ^†^	0.96
VEGF C (FI)	PA	91.78 ± 22.35	74.50 ± 20.29	**0.035** ^†^	0.76
VEGF D (FI)	PA	53.66 ± 16.09	30.57 ± 10.82	**<0.001** ^†^	0.99
IL-12p40 (FI)	PI	129.56 ± 45.91	81.64 ± 33.55	**0.003** ^†^	0.87
PEDF (FI)	GF	140.03 ± 32.88	121.21 ± 36.96	0.155 ^†^	0.29
EPO R (FI)	GF	85.50 ± 31.59	50.79 ± 17.64	**0.001** ^†^	0.94
IL-12p70 (FI)	PI	125.59 ± 45.28	71.43 ± 36.25	**0.001** ^†^	0.93
Kir 4.1 (OD)	NA	101.90 ± 5.60	98.99 ± 5.49	0.207 ^†^	0.27
AQP1 (pg/mg)	NA	153.64 ± 75.33	135.12 ± 59.44	0.474 ^†^	0.10
AQP4 (pg/mg)	NA	47.30 ± 21.27	39.31 ± 16.99	0.280 ^†^	0.18
AQP9 (pg/mg)	NA	360.64 ± 185,06	281.13 ± 86.31	0.144 ^†^	0.28
GFAP (pg/mg)	NA	69.77 ± 28.62	90.35 ± 35.13	0.093 ^†^	0.0003

SD: standard deviation; FI: fluorescence intensity; HC: Healthy Control; PI: proinflammatory; C: chemokine; GF: growth factor; PF: profibrotic; PA: pro angiogenic; NA: not applicable; IL-6: Interleukin-6; IL-8: Interleukin-8; MCP-1: Monocyte Chemoattractant Protein-1; GM-CSF: Granulocyte-Macrophage Colony-Stimulating Factor; MIP-3a: Macrophage Inflammatory Protein-3α; IL-12p40: Interleukin-12 Subunit p40; IL-12p70: Interleukin-12 p70; MIF: Macrophage Migration Inhibitory Factor; TGFβ-1: Transforming Growth Factor Beta-1; TGFβ-2: Transforming Growth Factor Beta-2; TGFβ-3: Transforming Growth Factor Beta-3; VEGF A: Vascular Endothelial Growth Factor A; VEGF C: Vascular Endothelial Growth Factor C; VEGF D: Vascular Endothelial Growth Factor D; MDC: Macrophage-Derived Chemokine; TARC (CCL17): Thymus and Activation-Regulated Chemokine (CCL17); CXCL16: C-X-C Motif Chemokine Ligand 16; FRACTALKINE (CX3CL1): Fractalkine (C-X3-C Motif Chemokine Ligand 1); GROa (CXCL1): Growth-Regulated Alpha Protein (C-X-C Motif Chemokine Ligand 1); PDGF-AA: Platelet-Derived Growth Factor-AA; PDGF-AB: Platelet-Derived Growth Factor-AB; PDGF-BB: Platelet-Derived Growth Factor-BB; GDNF: Glial Cell Line-Derived Neurotrophic Factor; PEDF: Pigment Epithelium-Derived Factor; EPO R: Erythropoietin Receptor; OPN: Osteopontin; Kir 4.1: Potassium Channel, Inward Rectifier 4.1; AQP1: Aquaporin 1; AQP4: Aquaporin 4; AQP9: Aquaporin 9; GFAP: Glial Fibrillary Acidic Protein. ^†^ Student *t*-test for independent samples. Significant *p*-values in bold. 95% confidence interval. Power values indicate the probability of correctly rejecting the null hypothesis (false positive rate) at a significance level of 0.05.

**Table 3:** Correlation between the stage of iERM and the concentration of cytokines found significantly higher when compared with controls.

Cytokine	Cytokine Primary Function	Correlation Coefficient
IL-6	PI	**0.85** ^†^
IL-8	PI	**0.82** ^†^
TARC (CCL17)	C	0.64 ^†^
FRACTALKINE (CX3CL1)	C	**0.84** ^†^
GDNF	GF	**0.87** ^†^
GM-CSF	PI	**0.86** ^†^
GROa (CXCL1)	C	**0.86** ^†^
MIF	PI	**0.81** ^†^
PDGF-AB	GF	**0.81** ^†^
PDGF-BB	GF	0.63 ^†^
TGF-β3	PF	**0.85** ^†^
TGF-β1	PF	0.78 ^†^
TGF-β2	PF	0.55 ^†^
VEGF A	PA	0.79 ^†^
VEGF C	PA	0.76 ^†^
VEGF D	PA	**0.89** ^†^
IL-12p40	PI	**0.87** ^†^
EPO R	GF	0.03 ^†^
IL-12p70	PI	**0.91** ^†^

^†^ Pearson’s correlation coefficient. Excellent correlation in bold. PI: proinflammatory; C: chemokine; GF: growth factor; PF: profibrotic; PA: pro angiogenic; IL-6: Interleukin-6; IL-8: Interleukin-8; GM-CSF: Granulocyte-Macrophage Colony-Stimulating Factor; IL-12p40: Interleukin-12 Subunit p40; IL-12p70: Interleukin-12 p70; MIF: Macrophage Migration Inhibitory Factor; TGFβ-1: Transforming Growth Factor Beta-1; TGFβ-2: Transforming Growth Factor Beta-2; TGFβ-3: Transforming Growth Factor Beta-3; VEGF A: Vascular Endothelial Growth Factor A; VEGF C: Vascular Endothelial Growth Factor C; VEGF D: Vascular Endothelial Growth Factor D; TARC (CCL17): Thymus and Activation-Regulated Chemokine (CCL17); FRACTALKINE (CX3CL1): Fractalkine (C-X3-C Motif Chemokine Ligand 1); EPO R: Erythropoietin Receptor; GROa (CXCL1): Growth-Regulated Alpha Protein (C-X-C Motif Chemokine Ligand 1); PDGF-AB: Platelet-Derived Growth Factor-AB; PDGF-BB: Platelet-Derived Growth Factor-BB; GDNF: Glial Cell Line-Derived Neurotrophic Factor.

## Data Availability

The data presented in this study are available in the article. Eventual additional data are available on request from the corresponding author.
